# Surgical Fluid Prescribing: When Are the Last Orders?

**DOI:** 10.7759/cureus.11765

**Published:** 2020-11-29

**Authors:** Robert A Bennett, George E Fowler

**Affiliations:** 1 General Surgery, Royal Devon and Exeter NHS Foundation Trust, Exeter, GBR

**Keywords:** junior, out-of-hours, fluid therapy, prescribing, general surgery

## Abstract

Introduction

Inappropriate fluid prescriptions result in excess morbidity and mortality in surgical patients. The majority of prescriptions are done by foundation year one doctors (FY1s) despite repeated evidence of poor knowledge and prescription habits among them when it comes to prescribing fluids.

Materials and methods

This was a retrospective observational study conducted at a 798-bed district general teaching hospital. Data for one year from an out-of-hours (OOHs) electronic task record system was extracted. An analysis was performed on all surgical ‘Fluid Reviews’ jobs recorded in the period from August 1, 2018, to August 7, 2019.

Results

During the 371-day study period, 1,283 requests for fluid reviews were made. Of these, 1,228 (95.7%) were assigned to the FY1 and 1,185 (92.3%) were requested by nurses. There was a mean of 3.5 ±2.1 requests per day. A bimodal distribution of requests was noted with peaks at 1900 and 2400. There was no discernible variation between different days of the week.

Conclusion

Fluid reviews were most frequently requested by nursing staff at times that coincide with their handover and the commencement of a new fluid chart at midnight. Reducing the number of inappropriate requests for fluid reviews may reduce the opportunity for inappropriate fluid prescribing. Improvements could be achieved through interventions in the ward rounds and by encouraging a multidisciplinary approach to education on fluid prescribing. Reducing the number of fluid prescriptions OOHs promotes continuity of care and education through patient follow-ups.

## Introduction

Inappropriate fluid prescribing has been well documented as a source of morbidity and mortality in surgical wards. Complications usually arise from regimes that are either too restrictive or too liberal [1-3], while the choice of fluid presents an additional challenge and remains a contentious topic [4]. The National Institute for Health and Care Excellence (NICE) has offered guidance regarding the same with clinical guidelines 174, ‘Intravenous fluid therapy in adults in hospital’ [5].

Considerable efforts have been made to understand the importance of appropriate fluid prescribing in the pre and intraoperative periods. However, fluid prescribing in the postoperative period is a topic that is less well researched [6]. The general advice is to wean fluids as soon as possible and use caution in patients following major abdominal surgery. Early cessation of intravenous fluids promotes the use of the gastrointestinal tract and encourages mobilisation [2,6].

Despite the complexities involved in fluid prescribing, the majority of prescriptions are performed by foundation year one doctors (FY1s) [1,7]. A survey of consultant surgeons in 2002 revealed that only 16% of respondents believed their pre-registration house officers were adequately trained and only 30% thought the fluids prescribed were adequate [8]. Subsequent studies have continued to show that FY1s have a poor knowledge base for fluid prescribing [9-14], with patients often given too much fluid with an excess of sodium and not enough potassium. Furthermore, fluid assessments are commonly performed without reference to the fluid balance or the latest biochemistry results [1,14-16].

Previous studies have focused on the type and quantity of fluids prescribed and the implications they have on patient morbidity [1,15]. They have consistently recommended improvement in junior doctor education [7,8,10,12-15], yet fluid prescriptions still remain highly variable [13,14]. This study explores the nature of fluid reviews during the out-of-hour periods (OOHs) to determine if additional improvements could be made by addressing how and when fluid reviews are requested.

## Materials and methods

This was a retrospective observational study conducted at a 798-bed district general teaching hospital in southwest England. General surgery and urology, including acute surgical admissions, manage 119 beds in the hospital. OOHs surgical ward cover and admissions, defined as 1700 to 0800 from Monday to Sunday, are covered by one FY1, one senior house officer (SHO), and one registrar. Handover for doctors is at 2030 and 0800 while handover for nurses is at 1930 and 0730.

Data were derived from an electronic task record system known as the ‘Doctor’s Taskboard’ (designed by Mr Karim Kamara, Application Development Manager, Royal Devon and Exeter Hospital). This software is used to request and assign jobs to doctors during the OOHs period. All requests must be set to one of 12 predefined job types. All jobs falling within one year of foundation training were extracted (August 1, 2018 0800 to August 7, 2019 0800). Duplicate and blank entries were removed. Tasks assigned to ‘Other’ were reassigned to ‘Fluid Reviews’ if appropriate. The resultant dataset was filtered to include all surgical ‘Fluid Reviews’ during the OOHs period prior to analysis.

This project was approved as a service evaluation study by the Clinical Audit Department at the Royal Devon and Exeter NHS Foundation Trust.

## Results

Over the 371-day (53-week) study period, 1,283 requests were made for fluid reviews in the OOHs period. The FY1 was assigned 1,228 (95.7%) of those, and the SHO was assigned 55 (4.3%). The colorectal, upper gastrointestinal, and urology wards made 518 (40.4%), 390 (30.4%), and 245 (19.1%) requests respectively. The remaining 130 (10.1%) reviews were requested in other wards. The majority of requests were made by nurses (1,185, 92.3%) with a small proportion requested by doctors (47, 3.7%). It is not known who requested the remaining 51 (4%).

**Figure 1:**
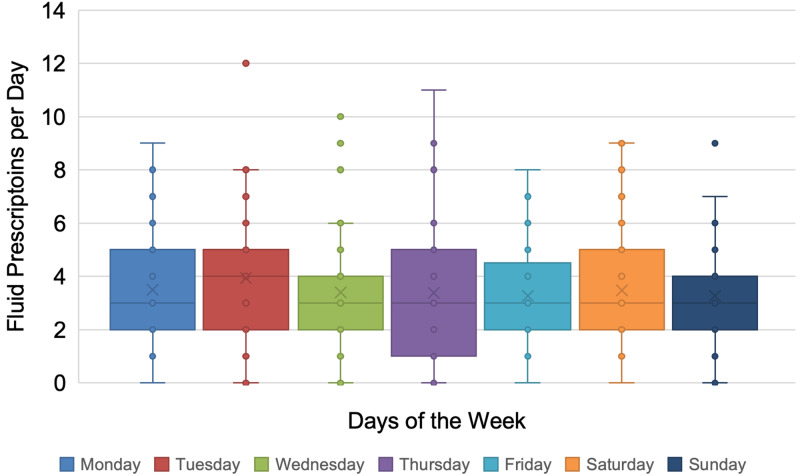
Distribution of surgical fluid reviews between 1700 and 0800

**Figure 2:**
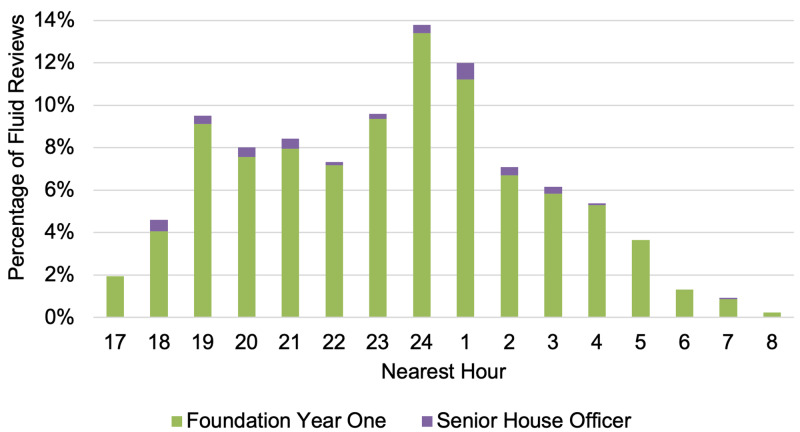
Distribution of fluid reviews throughout the out-of-hours period* *Time of request rounded to the nearest hour

**Figure 3:**
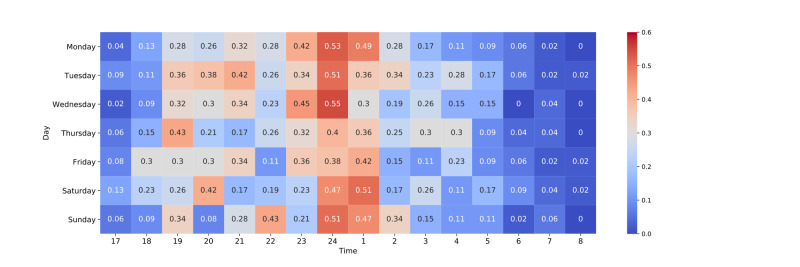
Mean number of surgical fluid reviews to the nearest hour in the out-of-hours period* *Results stratified by day of the week

Fluid reviews were uniformly requested OOHs Monday to Sunday with an average of 3.5 ±2.1 reviews per night (Figure 1). A bimodal distribution of fluid review requests was observed with peaks at 1900 and 2400. Nearly two-thirds of requests (67.5%) were logged between 2130 and 0800 with 49.8% falling between 2130 and 0230 (Figure 2). The average number of requests per hour remained consistent across the days of the week (Figure 3).

## Discussion

Nearly half of all fluid review requests were logged within a five-hour timeframe (2130 to 0230), with no discernible variation between different days of the week. Fluid reviews were commonly requested and peaked at 1900 and 2400, coinciding with nursing handover and the commencement of a new fluid chart at midnight. While a causal relationship could not be determined in this study, it suggests that the change of nursing staff and completion of the fluid balance chart may prompt the need for a fluid review.

Some fluid reviews may be unnecessary and result in the prescription of unwarranted fluids, particularly given fluid reviews are poorly performed by FY1 doctors [9-12,14]. In the immediate postoperative period, patients are encouraged to reduce their need for intravenous fluids by increasing their oral intake [2,6]. Those who are in the immediate postsurgery period would ideally have adequate and appropriate fluid prescriptions for the night prescribed by the anaesthetist, while patients with ongoing fluid requirements would have a detailed plan within the notes and prescriptions lasting until the morning if appropriate. Patients in need of more active fluid balance management should undergo further assessments before fluids are prescribed. The input of the anaesthetist is particularly important since urine output and oliguria may be unreliable indicators of hypovolaemia in the first 48 hours postoperatively [2]. Fluid reviews OOHs should therefore ideally only occur for patients with a change in clinical needs as identified by the nursing staff or for patients identified by the day team as requiring a scheduled fluid review. This may reduce the opportunity for erroneous prescribing following the lack of a detailed plan or possible omission by the day team.

Fluid reviews OOHs were predominantly requested by nursing staff with the majority completed by the FY1; this trend has not changed over the last decade [1,7]. Further research should assess both the appropriateness of nurse-led OOHs requests and the ability of FY1s to correctly identify which ones are appropriate. Interventions in the ward rounds may reduce the number of potentially unnecessary requests, while interventions targeting the practice of nurses may lead to earlier requests for fluid reviews by the day team. Both approaches would promote continuity of care and have the additional benefit of helping prescribing doctors consolidate their learning as they follow the effects that their prescribing have over subsequent days. This learning is difficult to establish when fluids are prescribed overnight.

This study has several limitations. Primarily, we used data from a single centre. However, this study identifies data for one year from a large district general hospital and may be generalised to other hospitals adopting a similar OOHs system. The study did not consider requests made during normal working hours, in-person or via the bleep system, and may have therefore underestimated the volume during this period. Nor did it assess how many reviews resulted in prescriptions and whether the prescriptions were appropriate or not. This study captures data representing 70% of the year by including times that fall outside of the normal working week (Monday-Friday, 0800-1700).

## Conclusions

Our study found that fluid reviews were commonly requested by nursing staff at times that coincide with their evening handover and the commencement of a new fluid chart at midnight. Foundation doctors need to be even more vigilant at these times, as fluids could be overprescribed in this time period. Interventions in the ward rounds and those involving nurses may reduce the number of referrals OOHs and subsequent opportunity for inappropriate prescription of fluids. Future studies could explore the appropriateness of fluid reviews OOHs and whether fluids are appropriately prescribed.
